# Application of Organoid Models in Prostate Cancer Research

**DOI:** 10.3389/fonc.2021.736431

**Published:** 2021-09-27

**Authors:** Ligui Zhou, Caiqin Zhang, Yongbin Zhang, Changhong Shi

**Affiliations:** ^1^ Animal Experiment Center, Guangzhou University of Chinese Medicine, Guangzhou, China; ^2^ Division of Cancer Biology, Laboratory Animal Center, The Fourth Military Medical University, Xi’an, China

**Keywords:** prostate cancer (PCa), organoid model, heterogeneity, pathogenesis, drug screening, individualized treatment

## Abstract

Complex heterogeneity is an important characteristic in the development of prostate cancer (PCa), which further leads to the failure of known therapeutic options. PCa research has been hampered by the current *in vitro* model systems that cannot fully reflect the biological characteristics and clinical diversity of PCa. The tumor organoid model in three-dimensional culture retains the heterogeneity of primary tumor tissues *in vitro* well and enables high-throughput screening and genome editing. Therefore, the establishment of a PCa organoid model that recapitulates the diverse heterogeneity observed in clinical settings is of great significance for the study of PCa. In this review, we summarize the culture conditions, establishments, and limitations of PCa organoids and further review their application for the study of pathogenesis, drug screening, mechanism of drug resistance, and individualized treatment for PCa. Additionally, we look forward to other potential developmental directions of PCa organoids, such as the interaction between prostate cancer tumor cells and their microenvironment, clinical individualized treatments, heterogeneous transformation model, tumor immunotherapy, and organoid models combined with liquid biopsy. Through this, we provide more effective preclinical experimental schemes using the PCa organoid model.

## Introduction

Prostate cancer (PCa) is one of the most common malignancies among men worldwide (1). In 2020, the incidence of prostate cancer in men was as high as 7.3%, second only to lung cancer. At the same time, the mortality rate reached 3.8% which led PCa to become the fifth major cause of cancer death in men (2). Since most PCa cases are androgen-driven adenocarcinomas, androgen deprivation therapy (ADT) is the main clinical treatment for early-stage PCa (3, 4). Most patients can benefit from treatment at an early stage through a rapid decrease in prostate-specific antigen (PSA) and a reduction in tumor volume. However, after a period of treatment, castration resistance ultimately ensues and the disease may develop into androgen receptor (AR)-castration-resistant prostate cancer (CRPC) or even neuroendocrine prostate cancer (NEPC), an AR-negative small cell neuroendocrine carcinoma (5, 6). The treatment for PCa is challenging due to its complex spatial, morphological, and genetic heterogeneity. Additionally, the oncologic transformation mechanism that results in clinical heterogeneity remains unclear. Therefore, there is an urgent need for prostate cancer preclinical models that can fully reflect the heterogeneity of PCa.

The research models used for the study of PCa mainly include traditional cell lines, conditional reprogramming cells (CRC), organoid models, patient-derived xenografts (PDX), and genetically engineered mouse models (GEMM). Different models have different advantages and disadvantages (**Table 1**). Traditional PCa cell lines, including LNCaP, VCaP, PC3, 22RV1, DU145, C4-2, and NCI-H660, are widely available and inexpensive. These cell lines show infinity growth, amenability to high-throughput screening and easy genome editing, but a lack of tumor heterogeneity and tumor microenvironment (7–9). In the CRC culture system, the combination of Rho kinase inhibitor Y-27632 and irradiated mouse fibroblast feeder cells enables primary cancer cells to acquire partial stem cell characteristics and the ability to indefinitely proliferate *in vitro* (10). However, androgen responsiveness of PCa is limited in this system and CRC is susceptible to contamination by feeder cells (11, 12). PDX, an important preclinical model *in vivo*, recapitulates tumor heterogeneity with high fidelity and correlates highly with patient responses (13). However, PDX is expansive and need a long time to establish (14). Most importantly, PDX is not amenable to high-throughput screening and genome editing (15). GEMM is spontaneous animal model that has been generated to emulate the expected functional consequences of key genomic alterations and has their own complete tumor microenvironment and immune system (16, 17). However, it is not only expensive and time consuming, but also only contains one or two genomic alterations by gene editing and is prone to induce multisystem tumors (18).

**Table 1:** Comparison of different prostate cancer model systems.

	Traditional cell lines (7–9)	CRC (10–12)	Organoid (20–25)	PDX (13–15)	GEMM (6–18)
Advantage	✧ Infinity growth ✧ Enable to High-throughput screening ✧ High availability and cheap ✧ Sample to genome editing	✧ High success rate of primary culture ✧ Enable to High-throughput screening ✧ Applied to PDX model ✧ Sample to genome editing	✧ Retain heterogeneity ✧ High-throughput screening ✧ Applied to PDX model ✧ Sample to genome editing ✧ Correlate with patient responses	✧ Retain heterogeneity ✧ Correlate with patient responses ✧ Low contamination of normal cell ✧ Include tumor microenvironment	✧ Spontaneous tumor ✧ Complete tumor microenvironment and immune system ✧ Generated nicely emulate the expected functional consequences of key genomic alterations ✧ Ease of genetic manipulation
Disadvantage	Lack of heterogeneity Lack of tumor microenvironment and immune system Low success rate	Lack of AR responsiveness Contamination of Feeder cells and normal cells	Low success rate of PCa culture No tumor microenvironment and immune system Contamination of normal cells Hard to long‐term propagation Contamination of and normal cells	Expensive and Time consuming Lack of immune system Low-throughput screening Low success rate of PCa culture Contamination of mouse cells	Expensive and Time consuming Murine PCa tumor Only one or two genomic alterations Induction of multisystem tumors

CRC, conditional reprogramming cells; PDX, patient-derived xenografts; GEMM, genetically engineered mouse models.

Therefore, a suitable model system, which can compensate for the shortcomings of the above-mentioned models, is particularly significant for PCa research. The organoid model might be such a compensatory model. Organoids in three-dimensional (3D) culture are derived from pluripotent stem cells or isolated organ progenitor cells to form organlike structures like the organs *in vivo* (19). Organoids encapsulate the diverse heterogeneity observed in clinical medicine (13, 14). More importantly, organoids are convenient for genetic manipulation and high-throughput drug screening *in vitro* (15, 16). Furthermore, they have a high correlation with the drug response of primary tumors in patients. On the other hand, PCa organoids also have some disadvantages, such as low efficiency of establishing organoids (particularly primary PCa), lack of microenvironment and immune system and the contamination of normal cells (17, 18). However, their unique advantages allow PCa organoids to be a great potential *in vitro* preclinical model, allowing for in-depth analyses of tumor heterogeneity.

In the present review, we review the culture conditions, establishments and limitations of PCa organoids and their applications in pathogenesis, drug screening, mechanism of drug resistance and individualized treatment for PCa. In addition, we further discuss the potential developmental directions of PCa organoids.

## Prostate Cancer Organoid Cultures

### Culture Conditions for the Development of PCa Organoid Models

**Figure 1:**
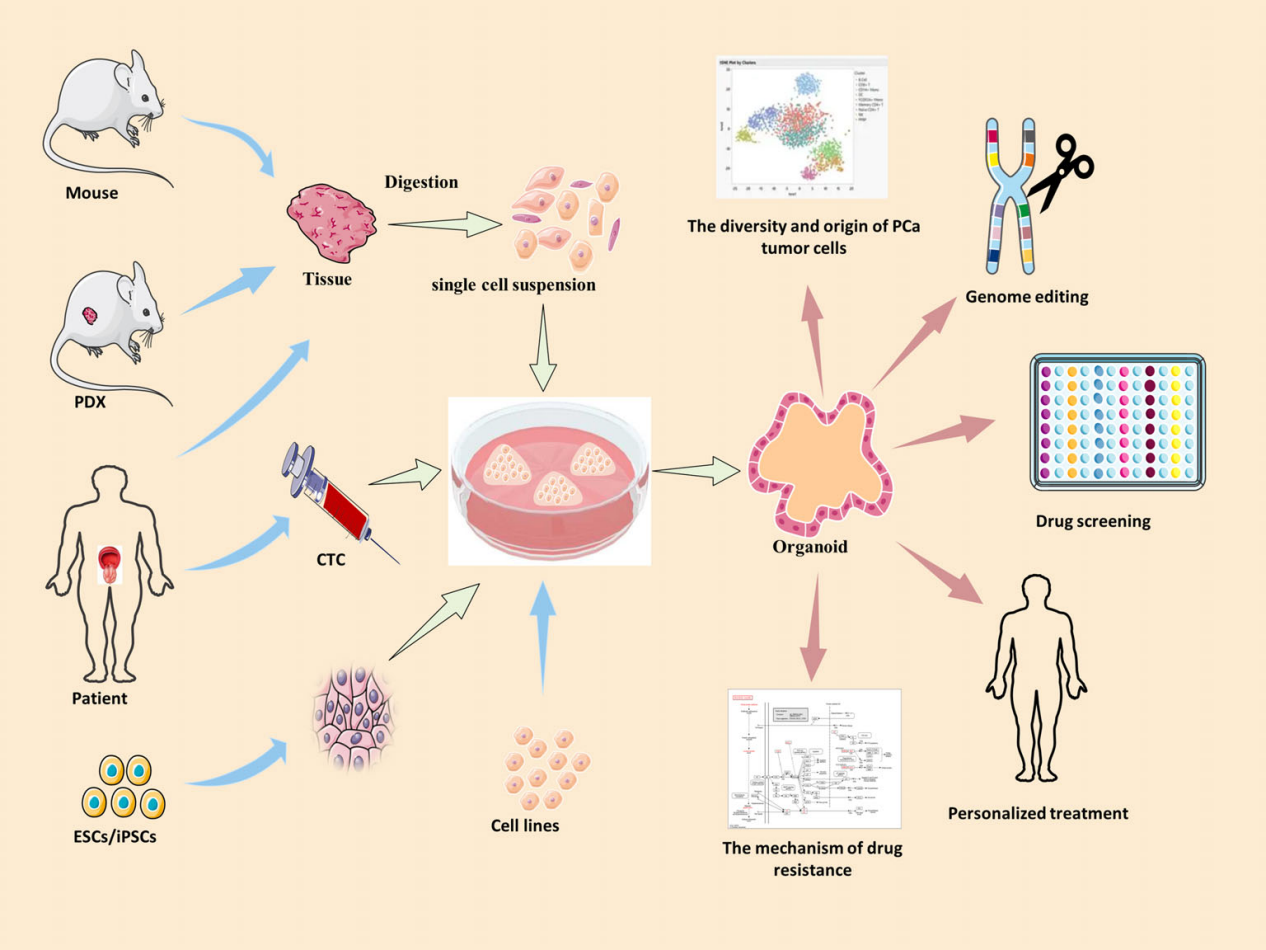
The summary of culture and applications of PCa organoid. Blue arrow →, the specimen source of PCa organoid. Red arrow →, the applications of PCa organoid. PDX, patient-derived xenografts; ESCs, human embryonic stem cells; iPSCs, induced pluripotent stem cells; CTC, circulating tumor cells.

The organoid model is a 3D culture of isolated pluripotent stem cells or organ progenitor cells in a matrix such as Matrigel. It provides a similar environment *in vitro* for cells or tissues to develop into micro-organs. The culture method of PCa has been described. **Figure 1** shows the general process of organoid culture for PCa. Sato et al. (26) first developed the universal organoid medium that contains advanced DMEM/F12 medium with epidermal growth factor (EGF), Noggin, and Wnt agonist R-spondin-1. On this basis, Drost et al. (27) continued to add different compounds and growth factors, including anaplastic lymphoma kinase (ALK) 3/4/5 inhibitor A83-01, dihydrotestosterone (DHT), fibroblast growth factor-10 (FGF10), fibroblast growth factor-2 (FGF2), prostaglandin E2 (PGE2), nicotinamide, and p38 inhibitor SB202190, N-acetylcysteine, B27 supplement and Rho kinase inhibitor Y-27632 to culture PCa organoids successfully (**Table 2**). Among them, growth factors FGF10, FGF2, PGE2, nicotinamide and SB202190 are not required when murine-derived PCa organoids are cultured. These organoids form a glandular structure, have a stable karyotype similar to that of the prostate *in vivo*, and complete AR signal transduction (28). In this culture system, PCa organoids can be successfully cultured within 2 weeks with an average split ratio of 1:2 every 2 weeks (27). Additionally, it can be applied to the PDX model, maintaining the heterogeneity of PCa (17). Based on these culture conditions, PCa organoids have been successfully established from normal tissues, tumor biopsy samples, circulating tumor cells (CTC), PCa cell lines, PDX models of PCa, human embryonic stem cells (ESCs) and induced pluripotent stem cells (iPSCs) (17, 27–32).

**Table 2:** Summary of culture medium components for mouse and human prostate organoids (27, 28).

Factor	Mouse organoids	Human organoids
Advanced DMEM/F12	pure	pure
HEPES	10 mM	10 mM
Glutamax	1x	1x
Penicillin/streptomycin	1x	1x
B27	1x	1x
Human epidermal growth factor	50 ng/ml	5 ng/ml
Human Noggin	100 ng/ml or 10% Noggin-conditioned medium	100 ng/ml or 10% Noggin-conditioned medium
Human R-spondin-1	500 ng/ml or 10% R-spondin-1-conditioned medium	500 ng/ml or 10% R-spondin-1-conditioned medium
A83-01	200 nM	500 nM
DHT	1 nM	1 nM
FGF10	–	10 ng/ml
FGF2	–	5 ng/ml
PGE2	–	1 μM
Nicotinamide	–	10 mM
SB202190	–	10 μM
N-acetylcysteine	1.25 mM	1.25 mM
Y-27632 dihydrochloride*	10 μM	10 μM

*Y-27632 dihydrochloride is only required in the culture medium after initial plating and passaging of the organoid using TrypLE.

DHT, dihydrotestosterone; FGF-10, fibroblast growth factor-10; FGF2, fibroblast growth factor-2; PGE2, prostaglandin E2.

Different growth factors play different roles in the culture of PCa organoids. R-spondin-1 and Noggin enhance the formation and expansion of organoids by promoting Wnt signaling and regulating the bone morphogenetic protein (BMP) signaling pathway, respectively (33, 34). Elimination of R-spondin-1 or Noggin will result in the inhibition of AR expression (28). Due to the high dosage and cost of R-spondin-1 and Noggin, the preparation methods of conditioned medium using modified cells for these factors are sorted in **Table 3** (27, 35, 36). EGF has been shown to be essential for prostate epithelial cell line growth derived from organoids, which vastly strengthens cell viability and proliferation (37). The absence of EGF upregulates AR signal transduction at the transcription and translation levels and also increases PSA expression (28). Previous research has suggested that EGF in culture medium could significantly reduce the sensitivity of PCa organoids to androgen resistance (38); thus, it is necessary to eliminate EGF during the pharmacological response of sensitivity to anti-androgen drugs. FGF2, PGE2, nicotinamide, B27 supplement, and transforming growth factor-beta (TGF-β) inhibitor A83-01 promote the proliferation of prostate cells to maintain the long-term growth of organoids (39–41). P38 kinase inhibitor SB202190 inhibits the histological keratinization phenomenon that may be increased by pressure signal transduction (28). DHT and FGF10 improve the efficiency of organoid formation (28). Rho kinase inhibitor Y-27632 can promote the proliferation of epithelial cells (42). It is worth noting that Y-27632 is only added to the medium during establishment of the first generation of organoids, after which there is no need to add it to the subsequent generations, and added when using TrypLE to digest organoids (27). N-acetylcysteine promotes organoid proliferation by inhibiting cell oxidation (43). These growth factors and hormones constitute the basic conditions to support the survival and long-term culture of PCa cells in the 3D culture system. These studies highlight the importance of media modification in culturing organoids. Required additive components differ depending on cell types, gene mutations and phenotypes of PCa. For instance, the addition of 10% FBS helps to promote the proliferation of PCa organoids for patients with bone metastases (44).

**Table 3:** Preparation of Noggin-conditioned medium and R-spondin-1-conditioned medium for organoids (27, 35, 36).

Medium	Cell line	Source	Preparation of culture medium
R-spondin-1-conditioned medium	293T-HA-Rspo-Fc cell line	Calvin Kuo Laboratory, Stanford University	Cells are cultured at ~75% confluence. Subsequently, the medium is replaced with advanced DMEM/F12 + 1 M HEPES + 1x Glutamax + 1x penicillin/streptomycin (AdDMEM/F12 +/+/+) for 1 week to create the conditioned medium.
Noggin-conditioned medium	293T/17 cell line	ATCC	pcDNA3.1-based mammalian expression vector containing mouse Noggin cDNA* is transiently transfected into a 293T/17 cell line. After transfection, the cells were cultured in AdDMEM/F12 +/+/+ for 1 week.

*pcDNA3.1-based mammalian expression vector containing mouse Noggin cDNA can be obtained from Hans Clevers Laboratory.

The culture of PCa organoids is still under continuous exploration and improvement. It has evolved from a separate organoid culture to a co-culture of organoid and cells related to the tumor microenvironment (45). For example, Richards et al. (46) co-cultured PCa epithelial cells with primary prostate stromal cells to study the direct interaction between epithelial and stromal cells. They found that the addition of stromal cells not only improved the ability to generalize tissue characteristics, but also improved the survival rate and formation efficiency of tumor organs. This indicates that organoid technology can successfully simulate the growth environment, *in vivo*, for tumor cells through co-culture with the tumor microenvironment. It can also provide a new platform for the study of the interaction of cancer cells with the microenvironment. Unfortunately, only the co-culture of PCa organoids with stromal cells has been studied, and co-culture studies with other tumor microenvironment cells have been reported.

### Establishment of Heterogeneous Organoid Models for Prostate Cancer

The specimen for organoid culture can be derived from any stage of tumor development and only a small piece of tissue or few cells are required to culture *in vitro* (47). Thus, different phenotypes of PCa organoids have been successfully cultivated. Karthaus et al. (28) used mouse prostate epithelial cells and human prostate specimens to cultivate normal prostate organoids composed of fully differentiated CK5^+^ basal cells and CK8^+^ luminal cells. It was found that such organoids retained the expression of AR signaling factors, prostate-specific transcription factor Nkx3.1, basal cell markers p63 and CK5, and luminal cell marker CK8. At the same time, the original tissues of mouse with *PTEN* knockout and *TMPRSS2-ERG* fusion gene were cultured in organoids, and the phenotypes were consistent. Additionally, Gao et al. (17) successfully cultivated seven metastatic castration-resistant prostate cancer (mCRPC)-type organoids from biopsy tissue specimens and CTC in patients with metastatic PCa. These organoids capsulate the molecular diversity and clinical heterogeneity of PCa subtypes, including *TMPRSS2-ERG* fusion, *ETS* translocation, *SPOP* mutation, *SPINK1* overexpression, *FOXA1* and *PIK3R1* mutation, *CHD1* deletion, *PTEN* deletion, and AR expression, and retain the genomic characteristics of primary tumors. These organoids reproduce a large repertoire of patient-derived CRPC lines with different genomic alterations to research PCa.

NEPC, a subtype of PCa with a poor prognosis, easily develops therapeutic resistance (48). Currently, there are only a few effective models for the study of neuroendocrine tumors. Puca et al. (49) successfully cultured four neuroendocrine-like PCa organoids from metastatic biopsy specimens, including simple small cell carcinoma and high-grade carcinoma with extensive neuroendocrine differentiation. They reported that the organoid model is a good tool for further understanding the characteristics of NEPC. Currently, androgen-sensitive and hormone-insensitive CRPC organoid models have been established, and CRPC transformation models have also been reported. Additionally, PCa cell lines LNCaP, C4-2B, and single luminal epithelial progenitors can also produce PCa organoids (29, 50). LNCaP produces androgen-dependent models while C4-2B produces a non-androgen-dependent model, each representing different clinical characteristics.

Recently, a novel *in vitro* modeling system combining the advantages of organoids and PDX has been reported (51). The PDX-organoid model takes less time and costs less than the PDX model and provides an adequate source of tissue for the establishment of organoid models. The LuCap PDX series used surgical specimens from metastatic PCa, successfully producing 21 PDX models (52). Beshiri et al. (30) produced PDX-organoid models with mCRPC characteristics similar to the LuCaP series. These PDX-organoid models retained the genomic heterogeneity of mCRPC and AR-dependent signaling, thus providing a good platform for the study of the pathogenesis, therapeutic options, and individualized treatment for mCRPC. Furthermore, bone metastatic prostate cancer (BMPC) organoid models derived from patients have been successfully established (44).

### Limitations of the Method in PCa Organoids

There are several limitations in the establishment of PCa organoids. Firstly, the overall success rate of PCa organoids is only 15-20% (17), limiting the extensive development of clinically diversified PCa models. A study produced statistics on organoids from 81 PCa specimens with diverse pathological and clinical features (53). The success rate of organoid development from metastatic prostatectomy reached 4/9 while that of organoid development from transurethral resection of the prostate was only 4/14. Another study reported a success rate of 16% (4/25) for organoids cultured from patients with metastatic PCa (49). This indicates that the key to the success of PCa organoids depends on the source and intrinsic characteristics of the sample. Therefore, it is necessary to improve the culture conditions for different origins or phenotypes of PCa specimens to obtain a high success rate. Secondly, the organoids from patients with primary PCa have not been successfully cultured to date. This is probably because tumor cells do not have a selective advantage over normal cells in the current culture medium (27). The optimal combination of culture factors still needs to be explored and verified repeatedly. Thirdly, the lack of availability of clinical samples of mCRPC patients hampers the establishment of a biological resource bank of PCa organoids that includes a wide variety of clinical phenotypes. Fourthly, it is still challenging to maintain the growth of organoids for a long time. Fifthly, PCa organoids contained only epithelial cells and/or stromal cells, and lacked some tumor microenvironment components, such as immune cells and vascular components. PCa organoids with an immune compatible microenvironment have not been developed. Therefore, they cannot be used for immunotherapy research.

## Application of Organoid Models in Prostate Cancer Research

As a major technological breakthrough, organoids have been recognized as an important tool for biomedical research. An existing study has shown that PCa organoids can not only recapitulate *in situ* histology *in vitro*, but also have a genetic mutational landscape similar to that of prostate cancer (17). This suggests that organoids provide a very representative *in vitro* model for the study of prostate cancer. Organoids allow research on disease tissue biology, mechanism of disease occurrence and development, drug screening, and personalized treatment in an environment that simulates endogenous cell tissue and organ structure (**Figure 1**). Therefore, we summarize the applications of prostate cancer organoids with respect to these aspects.

### Revealing the Diversity and Origin of Prostate Cancer Tumor Cells

*In vitro* models provide valuable insights into prostate biology, but current *in vitro* modeling systems are not representative of the cellular structure of the prostate. The organoid model better mimics prostate epithelial glands by recapitulating epithelial differentiation and cell polarity (50). Single-cell RNA Sequencing (RNA-Seq) analysis of patient-derived prostate epithelial cells revealed that compared to monolayer cultures, organoid cultures contained more distinct cell populations (54, 55). Ten cell types were identified *in vitro*, including a rare population of putative stem cells marked by high Keratin 13 (*KRT13*), Lymphocyte Antigen 6D (*LY6D*) and prostate stem cell antigen (*PSCA*). These results demonstrated that organoid culture condition has contribute to the survival and proliferation of different cell populations. It has allowed a deeper understanding of the cells present in prostate model systems and the creation of an in-depth atlas of the cellular population.

The origin cell of PCa remains a subject of debate. According to previous studies (56), PCa has two types of origin cells: basal cells and luminal cells. In tissue recombination models, only basal cells reconstitute a complete prostate gland (57, 58). However, it has been shown that luminal cells can generate basal cells through murine lineage-tracing experiments (59). Additionally, in the human prostate, only basal cells have been shown to be effectively transformed by select oncogenes (60). Organoid culture provides a unique model to solve this problem. After the organoid culture of basal and luminal cells of mouse/human prostate tissue were separated by fluorescence-activated cell sorting, both formed prostate-like organoids that retained both basal and luminal epithelial layers and preserved androgen-responsiveness in culture (28). This suggests that both basal and luminal cells have stem-like potential, and organoid culture can maintain stem cell characteristics of PCa. Another study (61) reported that c-MYC/myrAKT1-transduced human prostate basal- and luminal-derived organoids represented histological and molecular features of human PCa. These studies confirm that both human primary basal cells and luminal cells are the origin cells of PCa. Organoids can monitor the early development of PCa *in vitro* in real-time and directly compare the transformation of basal and luminal cells.

As is well-known, organoids are originally derived from cancer stem cells (CSCs) residing within the tumor bulk of the samples. PCa is a highly heterogeneous tumor harboring multiple cancer cell types. Different from the cell-of-origin that undergoes tumorigenic transformation due to gene mutation, CSCs are a cell population with self-renewal and pluripotent properties driving clonal tumor evolution (62). The existence of CSCs provides theoretical explanations for many molecular characteristics, cancer recurrence, metastasis and treatment resistance of PCa (63). Studies on CSCs have been hampered by the lack of suitable *in vitro* models. Here, organoids can be used as a good model *in vitro* for CSCs. Therapies targeting CSCs may lead to more effective cancer treatments for PCa. Therefore, organoid models can help us further understand the composition of normal stem cells and CSCs, which will be important in studying the occurrence and development of prostate cancer.

### Exploring the Key Genes Driving the Development of Prostate Cancer

The mechanisms that drive the pathogenesis of PCa and the series of clinical transformations are not well understood. Gene editing at the organoid level can simulate the effects of different gene mutations on the occurrence, development, and heterogeneous transformation of PCa *in vitro*. The easy handling of PCa organoid cultures *in vitro* also facilitates the editing of specific genes related to human diseases using lentivirus transfection, plasmid transformation, or the CRISPR/Cas9 gene-editing system (64, 65). Chua et al. (50) demonstrated that combined *PTEN* deletion and *Kras^G12D^* activation in organoids derived from CARNs (castration-resistant Nkx3.1-expressing cells) produced similar phenotypes to donor tumors of PCa *in vivo*. This proved that the deletion of *PTEN* and the routine mutation of *KRAS* are important in the induction of prostate tumors. The tumor origin cells and the chronological sequence of oncogenic events play an important role in defining the disease status. Using PCa organoids, Pietrzak et al. (66) proved that loss of the TIP5 transcription factor could trigger *PTEN*-loss mediated oncogenic transformation in prostate luminal cells, but becomes dispensable once the transformation is established. This suggests that TIP5-mediated chromatin states can control key developmental pathways and tumor suppressor genes, which could drive the development of cancer. In summary, transforming normal prostate organoids into cancerous organoids *in vitro* provides the optimal tool to identify the molecular subtypes of PCa in the genomic analysis of primary tumors.

Considering the heterogeneity of PCa, it is difficult to identify the true driver mutations. However, organoid technology displays a unique advantage. Because normal tissue-derived and tumor tissue-derived organoids from the same patient can be established simultaneously, the discrepancy in gene expression between the two can be compared to identify possible driver mutations. Furthermore, organoid modeling and gene editing can verify the screened mutant genes to study their influence on PCa. Normal tissue-derived organoid has relatively stable genetic information and can be used as a control model for studying tumor mutations.

### Drug Screening

Since PCa organoids can maintain genetic stability and heterogeneity, coupled with its easy high-throughput screening, it has become an ideal model for drug toxicity and efficacy evaluation (67). To explore whether CRPC-derived organoids were suitable for drug testing, they were used to determine the sensitivity to enzalutamide, everolimus, and BKM-120 (17). It was found that AR-amplified MSK-PCa2 organoid was extremely sensitive to enzalutamide and resistant to other drugs. Moreover, MSK-PCa2 organoid with *PTEN* loss and *PIK3R1* mutation was sensitive to everolimus and BKM-120, consistent with the results *in vivo*. This result demonstrates the utility of CRPC-derived organoids in assessing drug sensitivity, similar to findings in clinical trials. Further, these results suggest that organoids can be used for drug screening and modeling individualized treatment. Four CRPC-neuroendocrine (CRPC-NE) organoids and two CRPC-adenocarcinoma (CRPC-Aden) organoids were established for high-throughput sequencing of 129 chemotherapeutics and targeted drugs (49). The results demonstrated that the AR antagonist, enzalutamide and taxane chemotherapies, cabazitaxel and docetaxel were effective for CRPC-Aden organoids. A small number of drugs, such as pozotinib and vandetanib, were found to have significant activity in CRPC-NE organoids.

Organoid models are effective *in vitro* drug testing platforms for identifying potential pharmacological treatments and screening inhibitors targeting different phenotypes of PCa. For instance, Jansson et al. (68) screened 110 drugs from CRPC LuCaP PDX-derived organoids and showed that HSP90 inhibitors had a significant inhibitory effect on CRPC. Further studies have demonstrated that the HSP90 inhibitor, ganetespib, decreased tumor growth by inhibiting multiple targets including the factors involved in AR and PI3K pathways. Using established PTEN/TP53 null LuCaP 136 tumors, they found that compared with any single therapy, the combination of ganetespib and castration significantly inhibited tumor growth and led to a delay in castration resistance.

### Studying the Mechanism of Drug Resistance

Gene mutation, chromosome amplification, and chromosome rearrangement are the main reasons for the development of drug resistance in tumors (69). Patient-derived organoids (PDO) of drug resistance can be established according to the different mutations in patients to explore targets for improving the prognosis of patients with PCa, which is incomparable with existing PCa cell lines. It has been confirmed that there are at least three general mechanisms for the development of resistance in CRPC (38). First, genetic mutations, such as *AR*, *ETS*, *TP53*, and *PTEN* gene mutations, lead to the activation of the AR signaling pathway (70). Second, the activation of bypass signals, such as the glucocorticoid receptor pathway, compensates for the loss of the AR signal (71). Third, during treatment, tumor cells acquire resistance by switching lineages from a cell type, dependent on the drug target, to another, which is not. This may be represented by cases of PCa that are AR-negative or neuroendocrine specific (72, 73). The corresponding heterogeneous organoid models will represent their respective resistance mechanisms. Moreover, to clarify the molecular mechanism of PCa resistance, some groups have used PCa organoids as a platform for rapid detection of the effects of different mutations on anti-tumor drugs using CRISPR/Cas9 or lentivirus transfection technology for gene editing. For instance, Pappas et al. (38) used the PCa organoid model to demonstrate that the loss of *p53* did not induce resistance to androgenic molecules but *Pten* deficiency increased resistance to androgenic drugs. However, the dual loss of *p53* and *Pten* resulted in complete resistance to the second generation of anti-androgen drugs. Dai et al. (74) found that PCa-associated *SPOP* mutations conferred resistance to bromodomain and extra-terminal (BET) inhibitors by stabilizing bromodomain-containing protein 4 (BRD4). The development of resistance and relapse in CRPC tumor cells is a major problem in the field of PCa research. Since 3D organoid cultures simulate the emergence of treatment-resistant residual tumors, it provides a more effective platform for study. Dhimolea et al. (75) reported that treatment-resistant residual tumor cells in organoids, xenografts, and cancer patients entered a dormant diapause-like adaptation to reduce apoptosis priming by suppressing *MYC* activity or inhibiting *MYC* transcriptional co-activator *BRD4*, which could weaken drug cytotoxicity and induce the tumor to be resistant to treatment. Therefore, organoid culture can be useful in constructing a heterogeneous PCa model *in vitro* to study the influence of specific genes on PCa resistance with the help of gene-editing technology. It is beneficial for developing first-line therapeutic drugs or screening new therapeutic targets.

### Personalized Treatment

Organoids, as preclinical cancer models, can be used to achieve precision medicine through *in vitro* therapeutic screening of individual patient samples. Several studies have demonstrated that drug response to organoids may predict clinical outcomes (76, 77). Although many clinical trials related to PDO models are carried out, there are limited reports about PCa. Beltran H’s team cultured CRPC-NE samples from patients into organoids for high-throughput drug screening. One CRPC-NE organoid, OWCM155, showed significant sensitivity to aurora kinase inhibitor alisertib (49), a result concordant with what was being observed from the clinical response of corresponding patients in the phase II trial of alisertib for CRPC-NE (NCT01482962) (78). On the other hand, the CRPC-NE organoid, OWCM154, did not react to alisertib *in vitro*, nor did the patient in phase II clinical trial. In additional, Beshiri et al. (30) found that LuCaP-derived organoids with *BRCA2* deficiency were sensitive to olaparib, which was consistent with the response observed in clinical setting (79). These data indicate that organoids may be useful tools as patient ‘avatars’ for clinical trials and applied to develop new strategies for precision medicine in cancer. However, not all drug responses of organoids are consistent with clinical responses. Karkampouna at al. (80). cultured PDOs for drug screening. The organoids from an advanced PCa patient (case P82) as well as from a primary PCa patient (case P134) were both resistant to enzalutamide *in vitro*, while cases P82 and P134, were sensitive and resistant, respectively, to enzalutamide. The correlation between drug responses of organoids and clinical responses may depend on the size or site of the patient sample. Larger sample sizes would be needed to increase the confidence level in the data. The high heterogeneity of PCa and the drug profiles correlated with disease stage, sometimes leads to incompatibility between the results of the drug response in organoids and the clinical response of the patient.

## Discussion

As a novel *in vitro* preclinical disease model, the PCa organoid has potential for broad applications. This model is not only convenient for *in vitro* applications, but also demonstrates genetic stability and heterogeneity. Not only can it be applied for multi-model verification of PCa pathogenesis and drug screening combined with the PDX animal model, but also for observing the growth and development of tumor cells or tissues *in vitro* and studying the effects of different mutations on the occurrence and development of diseases. This model provides effective guidance for studying the heterogeneous transformation mechanism of PCa, especially to understand the molecular mechanisms underlying androgen resistance and to screen potential therapeutic targets. From the perspective of drug screening and individualized treatment, different primary or metastatic PCa organoids can be established using PCa samples from different sources. Combined with high-throughput screening technology, anti-PCa drugs can be assessed in batches. The response of patients with PCa to different drugs can be predicted effectively, which will accelerate the development of therapeutic regimens for heterogeneous characteristics of PCa. In PCa organoids, CRISPR/Cas9 and shRNA techniques have been used to study the mechanism of drug resistance. This will allow the study of the functions of drug resistance genes, and will ultimately enable the improvement of first-line therapeutic drugs and the development of novel individualized drugs. Inevitably, PCa organoids still have some challenges including low efficiency of establishing organoids (particularly primary PCa) from human samples, the optimal combination of medium factors that need to be added and the maintenance of culturing those organoids for a long time. Additionally, there are new application directions for PCa organoids.

### Exploring the Interaction Between Prostate Cancer Tumor Cells and Their Microenvironment

Exploring the interaction between the tumor and its microenvironment is an important aspect of oncology research. Organoid culture of the vascular system *in vitro* simulates the interaction between tumor cells and the vascular system while that of the nervous system revealed the interaction between cells and the nervous system. Immunocytes are co-cultured with tumor organoids to reveal the relationship between tumor cells and immune infiltrating cells (81). It is necessary to further study the role of fibroblasts, immune cells, and endothelial cells in the tumor microenvironment so that the structure of PCa organoids can better represent the composition of PCa *in vivo*.

### Clinical Individualized Treatments for Patients With Prostate Cancer

The lack of *in vitro* tumor models maintaining the characteristics of tumor cells *in vivo* has become a bottleneck in the realization of personalized therapy and precision therapy for patients with cancer. Organoids are an ideal model for drug testing and screening because of their ability to be cultured *in vitro* for a long time to maximize the characterization of tumor cells *in vivo*. Therefore, to better reflect the clinical heterogeneity of PCa, it is necessary to improve the culture conditions in the future to better support the growth of different types of PCa cells. In 2014, world’s first prostate tumor organ bank was established (18). These organoids were used to test a variety of experimental drugs and cancer drugs, finding that tumor organs with different genetic backgrounds had different sensitivities to these drugs.

### Heterogeneous Transformation Model

Patients with CRPC that have high treatment selection pressure can lead to a significant increase in PCa heterogeneity (82), such as changes in AR and PSA expression levels, AR mutations, and the occurrence of the NEPC phenotype. Therefore, more CRPC models are needed to represent different resistance mechanisms, especially dynamic models to reflect their transformation characteristics. The existing organoid models only reflect the static characteristics of a patient at a certain stage and are insufficient in simulating the diversity and disease progression of PCa. Therefore, it is inevitable to develop a model based on the same specimen that can be induced to reflect the whole process of PCa transformation, including the development of CRPC, the transformation from adenocarcinoma to NEPC, and the occurrence of metastasis. This aids tremendously in investigating progression from pre-neoplastic to neoplastic to metastatic states. Additionally, a paired comparison model can be generated through organoid culture to compare genotypes and phenotypes and explore the mechanism of their heterogeneity.

### Tumor Immunotherapy

The application of tumor organoids in tumor immunology offers researchers another attractive preclinical option. Studies (83–85) have found that tumor organoids can be co-cultured with immune cells isolated from autologous tumor tissues or peripheral blood of healthy donors. Further studies on different immune cells should be performed, including amplification of tumor organoids *in vitro* to explore the interaction between the immune system and tumors. In the model of tumor immunotherapy, compared with immortalized tumor cell lines and humanized immune-oncology models (86, 87), the co-culture of tumor organoids and immune cells from tumor tissues better represents the response of patients to immunotherapy. The co-culture system of tumor organoids and immune cells has a short experimental cycle and does not have the problem of human-mouse immune compatibility, which is expected for an ideal model for tumor immunotherapy.

### Organoid Models Combined With Liquid Biopsy

The patient derived PDX model of PCa was established using a specimen from surgical resection or autopsy, highlighting the need for more specimens. At the same time, the heterogeneity within the tumor is one of the challenges of traditional tissue biopsy. Liquid biopsy is expected to overcome these issues. CTC can be used as an alternative to invasive biopsies to study the heterogeneity of tumors (88). However, liquid biopsy has disadvantages in the detection and enrichment of CTC since only a few CTC can usually be obtained. Tumor organoids can be grown from only limited amounts of specimens to solve this problem. Gao et al. (17) confirmed the feasibility of using a small amount of CTC from peripheral blood of patients with PCa to culture tumor organoids. Exome sequencing showed that PCa organoids derived from CTC retained molecular diversity consistent with primary tumors, including *TMPRSS2-ERG* fusion, *SPOP* mutation, *SPINK1* overexpression, and *CHD1* loss. This is the only successful case of derivation of organoids from blood samples reported thus far. Liquid biopsy combined with tumor organoids may create new opportunities for minimally invasive studies and may facilitate the inclusion of PDO in personalized medical procedures for cancers.

In conclusion, it is necessary to continuously optimize the culture conditions of PCa organoids, constructing a tumor microenvironment more suitable for the different mutations in PCa. Further improving the success rate of PCa organoids is beneficial for obtaining a wider range of phenotypes and genotypes. This will allow the construction of models for hormone naïve PCa, CRPC, and mCRPC to fully demonstrate the clinical heterogeneity of PCa. This will also allow the formation of a PCa organoid bank to maximize its utility in mirroring clinical characteristics and become the preferred preclinical disease model of PCa.

## Author Contributions

LZ, YZ, and CS: conceptualization. LZ, CZ, and CS: writing. LZ, CZ, and CS: revising. All authors contributed to the article and approved the submitted version.

## Funding

This study was funded by the National Natural Science Foundation Program of China (No. 31772546 and 32070532) and Laboratory Animal Foundation Program No. SYDW[2017]-02.

## Conflict of Interest

The authors declare that the research was conducted in the absence of any commercial or financial relationships that could be construed as a potential conflict of interest.

## Publisher’s Note

All claims expressed in this article are solely those of the authors and do not necessarily represent those of their affiliated organizations, or those of the publisher, the editors and the reviewers. Any product that may be evaluated in this article, or claim that may be made by its manufacturer, is not guaranteed or endorsed by the publisher.

## Abbreviations

PCa, prostate cancer; ADT, androgen deprivation therapy; PSA, prostate-specific antigen; AR, androgen receptor; CRPC, castration-resistant prostate cancer; NEPC, neuroendocrine prostate cancer; CRC, conditional reprogramming cells; PDX, patient-derived xenografts; GEMM, genetically engineered mouse models; 3D, three-dimensional; EGF, epidermal growth factor; ALK, anaplastic lymphoma kinase; DHT, dihydrotestosterone; FGF10, fibroblast growth factor-10; FGF2, fibroblast growth factor-2; PGE2, prostaglandin E2; CTC, circulating tumor cells; ESCs, embryonic stem cells; iPSCs, induced pluripotent stem cells; BMP, bone morphogenetic protein; TGF-β, transforming growth factor-beta; mCRPC, metastatic castration-resistant prostate cancer; BMPC, bone metastatic prostate cancer; RNA-Seq, RNA Sequencing; KRT13, Keratin 13; LY6D, Lymphocyte Antigen 6D; PSCA, prostate stem cell antigen; CSCs, cancer stem cells; CARNs, castration-resistant Nkx3.1-expressing cells; CRPC-NE, CRPC-neuroendocrine; CRPC-Aden, CRPC-adenocarcinoma; PDO, patient-derived organoids; BRD4, bromodomain-containing protein 4.
